# Feeding Ration Impacts Larval *Pimephales Promelas* 7-Day Subchronic Growth Endpoint: Case Study with Perfluorooctanesulfonic Acid

**DOI:** 10.1007/s00244-024-01068-8

**Published:** 2024-05-25

**Authors:** Jonna Boyda, David Moore, Paige Krupa, Ashley Kimble, Thomas Biber, Lauren May, Alan Kennedy

**Affiliations:** https://ror.org/027mhn368 grid.417553.1 0000 0001 0637 9574 U.S. Army Engineer Research and Development Center, Environmental Laboratory, 3909 Halls Ferry Road, Vicksburg, MS 39180 USA

## Abstract

The larval fathead minnow, *Pimephales promelas*, 7-day subchronic survival and growth standard toxicity test method is commonly used for research and regulatory testing of effluents and compounds, including emerging contaminants such as Perfluorooctanesulfonic Acid (PFOS). Existing feeding guidelines for testing are described in multiple methods but are open to interpretation. The current study sought to determine the impact of feeding ration on *P. promelas* survival and biomass during a subchronic exposure to PFOS. The study was conducted in two phases: (1) a control experiment to determine the most significant feeding ration factors that maximize biomass, with consideration to laboratory logistics, and (2) application of down-selected feeding rations in a PFOS exposure to determine toxicity reference values. The control optimization study supported that feeding ration and feeding frequency were significant factors in fish biomass. In the subsequent PFOS study, fish were fed a high or low ration of *Artemia* twice daily, while exposed to 0.3 to 3.4 mg/L PFOS. Fish fed a high ration of *Artemia* had significantly (*p* < 0.05) greater biomass than fish fed a low ration in all exposure concentrations except 3.4 mg/L, where survival was low in both treatments. The feeding ration was not a significant factor on the survival endpoint for either treatment, but the PFOS concentration was (*p* < 0.0001) (high ration LC_50_ = 2.44 mg/L; low ration LC_50_ = 2.25 mg/L). These findings contribute to a better understanding of the impact feeding ration has in toxicity assessments and downstream regulatory decisions.

**Supplementary Information:**

The online version contains supplementary material available at 10.1007/s00244-024-01068-8.

Aquatic toxicity tests using model fish and invertebrate species are commonly employed to determine toxicological thresholds. Toxicity testing is useful for assessing the potential hazard of field collected effluents, surface waters or specific chemicals, such as the emerging contaminant perfluorooctanesulfonic acid (PFOS), a type of per—and polyfluoroalkyl substance (PFAS). PFAS are a group of synthetic chemicals that were widely used in industrial and consumer products such as flame retardants, food package coatings, non-stick cookware, and stain repellants (Trier et al. 2011; Sontake and Wagh 2014; Mohamed et al. 2019). These compounds, referred to as “forever chemicals” are widespread in aquatic environments and are a cause of concern because of their (1) long environmental persistence, (2) ability to bioaccumulate in a food chain, and (3) negative human health implications (Trier et al. 2011; Macorps et al. 2022; Barbo et al. 2023; Seyyedsalehi and Boffetta 2023). With more than 15,000 forms of PFAS, we chose to examine PFOS in this study because it is one of the most common compounds found in the environment, and due to evidence suggesting its higher aquatic toxicity compared to perfluorooctanoic acid (PFOA) (Hayman et al. 2021). Multiple studies have documented reproductive and developmental toxicity thresholds of PFOS at multiple life stages of *P. promelas*, the fathead minnow, furthering knowledge of potential adverse impacts, but there are research questions that remain unanswered (Ankley et al. 2005; Oakes et al. 2004; Suski et al. 2020; Bartlett et al. 2021). The study herein contributes to the knowledge gap by characterizing growth and survival impacts of PFOS at a critical life stage. Furthermore, it continues extensive PFOS research in our laboratory and it was recently suggested that fish testing methodologies may have substantial impacts on regulatory values for PFAS (Krupa et al. 2022; Gust et al. 2023).

One of the critical aspects addressed in toxicity tests is the assessment of growth, or biomass, as an assessment endpoint. However, fish biomass may be influenced by more than chemical toxicity; the type of food, total amount of food provided, and frequency of feeding all impact growth of various species, but particularly larvae and fry life stages (Lall and Tibbetts 2009; Aderolu et al. 2010; Kasiri et al. 2011; Kikuchi et al. 2006). Several organizations publish standard procedures for toxicity testing, specifically for *P. promelas*, including the U.S. Environmental Protection Agency (USEPA 2002a, 2002b), Environment Canada (2011), Organisation for Economic Co-operation, and Development (OECD) Guidelines for the Testing of Chemicals (OECD 2012a) and the American Society of Testing and Materials (ASTM 2022). These protocols ensure that tests are carried out consistently at various worldwide laboratories.

*Artemia* nauplii (brine shrimp) are the recommended food for *P. promelas* testing in published guidelines (USEPA 2002a; 2006, ASTM 2022, OECD 2012a, Environment Canada 2011). Multiple USEPA protocols suggest feeding larvae 0.1 g *Artemia* three times daily, at 4-h intervals, or 0.15 g twice daily, at 6-h intervals (USEPA 2002a, 2006). It is not specified if the 0.1 or 0.15 g is wet or dry biomass, but the USEPA estimates that 0.1 g is approximately 700 to 1000 individual organisms (USEPA 2006). The Agency also stated that “sufficient numbers” of nauplii should be supplied to ensure that there is extra [food] available for hours afterward, but not so much that water quality is degraded (USEPA 2002a). The language used in USEPA 2002a and 2006, specifically with regards to “sufficient numbers” and “concentrated” when referring to excess *Artemia,* is unspecific and may lead to variable interpretations among labs and technicians. Furthermore, the use of marine-adapted *Artemia* in these tests may introduce complications since these organisms can decay within a relatively short period (4–8-h) when placed in freshwater bioassay chambers (USEPA 2002b). It then becomes imperative to provide multiple feedings daily to ensure a constant supply of *Artemia* to the test organisms, which typically do not consume dead *Artemia,* as recommended by established protocols (USEPA 2006; Environment Canada 2011). Lab workers may underfeed larval fish due to water quality concerns, inadvertently confounding the biomass endpoint and subsequent data and analyzation.

Although not specifically referring to the 7-day subchronic toxicity test for *P. promelas,* the OECD (2013) guidance for early-life stage toxicity testing with fish acknowledges there are opportunities for continual revisions to improve survival and optimize biomass in larval tests. The OECD (2013) guidance advises that newly-hatched larval *P. promelas* be fed *Artemia*, but there is no suggested frequency or specific amount (i.e., “the correct food for each stage should be supplied from an appropriate time and at a level sufficient to support normal growth”) and any excess should be removed to avoid excess waste. An ASTM (2022) method on early-life stage toxicity tests recommended the same feeding frequency intervals as the USEPA documents (USEPA 2002a, 2006). ASTM (2022) also stated that there must be multiple feedings at least 5 days a week and once on all other days (e.g., weekends). These guidelines support traditional ad libitum (as much or as often as necessary or desired) feeding methodologies to allow flexibility for testing by a wide array of workers, laboratories, and scenarios. However, ad libitum guidance for bioassays may be unclear and interpreted inconsistently depending on experience level. Individual interpretations impact toxicity testing outcomes and risk management decisions, by allowing researchers to conveniently fit two to three feedings in within an 8-h workday and once per day on weekends. However, it is not clearly indicated when to start a test in relation to the once daily weekend feedings, and if said feeding at the beginning, middle or end of the day is preferable to optimize biomass.

The overarching goal of this study was to generate data to support future consideration of a more prescriptive feeding ration protocol for the 7-day *P. promelas* survival and growth test method that offers clarity, performance, and consistency, with consideration to laboratory and workday logistics. Because toxicity data generated for legacy and emerging contaminants informs risk assessments and regulatory decisions, we wanted to ensure good laboratory practices are employed in a precise, consistent, and repeatable manner by detailing and recording feeding methodologies. Specific objectives were to: (1) analyze potential interactions between the independent factors of fish density, total *Artemia* provided, and feeding frequency associated with fish biomass, (2) determine which combination of independent factors supports robust biomass, and (3) test the new methods during an exposure study with PFOS, a relevant emerging contaminant. The present study is the first the authors are aware of that rigorously evaluates the effect of the total food provided and frequency of feeding for the 7-day subchronic *P. promelas* test method, with consideration of a PFOS chemical exposure.

## Materials and Methods

### Experimental Design

*Pimephales promelas* (< 24-h old embryos) were shipped overnight from Aquatic BioSystems (Fort Collins, Colorado) and the larvae received were acclimated in 5-gallon glass tanks (Fisher Scientific, Waltham, Massachusetts), containing moderately hard reconstituted water (MHRW). Water was made according to USEPA (2002a), in an environmentally controlled walk-in chamber (Darwin Chambers, St. Louis, Missouri) at 25 ± 1 °C. Larvae were held overnight until test initiation the next morning and were within the age limit (< 48-h old), allowed by USEPA (2002a, 2006) guidelines when organisms are shipped. Feeding experiment procedures were adapted from those outlined by USEPA (2002a) for the 7-day larval growth method. Experiments were conducted in 600 mL Pyrex glass beakers containing 450 mL of MHRW (for control feeding study) and PFOS solution (for exposure study) throughout the 7-day test duration. An 80% water change that removed waste and excess food was completed daily (± 1 h) from days 1 through 6 as per USEPA (2002a). Water quality parameters (e.g., temperature, dissolved oxygen, pH, and conductivity) were recorded before and after each water change using the YSI Professional Plus (Pro Plus) Multiparameter Instrument (SKU 6050000) and Pro Series Quatro Field Cable (SKU 605790–1) according to manufacturer instructions (YSI Incorporated, Yellow Springs, Ohio). Briefly, there were no extremes of temperature (23.6–24.6 °C), dissolved oxygen (4.27–9.26 mg/L), pH (6.93–8.29 SU), and conductivity (270.7–305.2 μS/cm) in the test vessels, consistent with acceptability criteria in the USEPA test methods (USEPA 2002a). A full report of water quality parameters is available upon request.

### Control Study

A total of 270 fish were randomly assigned across 12 experimental combinations, tested in triplicate. Fish were allocated into three groups (e.g., Group 1, Group 2, Group 3), with each group having 4 different treatments (Table 1). Briefly, Group 1 was fed 3 times daily at intervals of 6-h (e.g., 8am, 2pm, 8pm). Group 2 was fed 2 times daily at 12-h intervals (e.g., 8am, 8pm). Group 3 was fed 2 times daily at 6-h intervals (e.g., 8am, 2pm). Furthermore, 2 treatments from each group had a “high” density of 10 fish per beaker or a “low” density of 5 fish per beaker, to determine the impact on growth when food resources were less limited, and to potentially determine any reductions on water quality. The lower density was employed to observe food resource limitations relative to the higher density, not to investigate alternative fish loading numbers for the standardized 7-day subchronic USEPA method. Each density was fed a “high” ration or a “low” ration of *Artemia* (2000 or 1000 individuals for high density and 1000 or 500 for low density).

**Table 1:** Control feeding study experimental design notation by group, with treatments outlined. Each treatment consisted of 3 replicates. Final fish survival counts are displayed and were used in calculating average weights in statistical analyses

	# fish	# feedings/day time	# *Artemia*/beaker/day	Nomenclature*	Ratio: # *Artemia*/ # fish (daily)	Ratio: # *Artemia* /feeding/#fish	Final #fish alive
Group 1	10	3 8am/2pm/8pm	2000	10F-3X(6H)-2000	200	67	29/30
	5		1000	5F-3X(6H)-1000	200	67	14/15
	10		1000	10F-3X(6H)-1000	100	33	30/30
	5		500	5F-3X(6H)-500	100	33	15/15
Group 2	10	2 8am/8pm	2000	10F-2X(12H)-1000	200	100	30/30
	5		1000	5F-2X(12H)-1000	200	100	15/15
	10		1000	10F-2X(12H)-1000	100	50	30/30
	5		500	5F-2X(12H)-500	100	50	15/15
Group 3	10	2 8am/2pm	2000	10F-2X(6H)-2000	200	100	30/30
	5		1000	5F-2X(6H)-1000	200	100	15/15
	10		1000	10F-2X(6H)-1000	100	50	30/30
	5		500	5F-2X(6H)-500	100	50	15/15

^*^Notation defines fish density (10 fish or 5 fish); feeding regime (2 times daily with a 6- or 12-h interval or 3 times daily with a 6-h interval); high or low feeding ration of 1000 or 500 *Artemia* nauplii

### PFOS Study

A total of 600 fish were used in this phase of the study, split among two treatments: a high feeding ration (HFR) (2000 *Artemia* nauplii per day) and a low feeding ration (LFR) (1000 *Artemia* nauplii per day). Both feeding rations were provided twice daily, at 8am and 5pm. Two feedings daily at those specified times allowed for laboratory technicians to complete the task within business hours and ensured there was live *Artemia* available into the evening. There were 5 exposure concentrations and 1 control in each treatment, all with 5 replicates and 10 fish in each.

### PFOS Solutions

PFOS was purchased from Sigma-Aldrich (CAS no. 2795–39-3; > 98% purity; Product #: 77,282, Lot #: BCCC4690, St. Louis, Missouri). A preliminary range finder was performed to determine the nominal concentrations that would allow for adequate survival of the larval fish during the subchronic exposure. PFOS test solutions were made by dissolving a nominal mass of 5 mg/L in MHRW via inversion of a volumetric flask, for the highest concentration. Additional concentrations were mixed by dilution with MHRW, in a 50% dilution series (nominal values were 0.312, 0.625, 1.25, 2.5, and 5 mg/L). Water samples were collected to obtain measured PFOS exposure concentrations: (mean ± SD) 0.003 ± 0.004 (control), 0.31 ± 0.009, 0.64 ± 0.3, 0.89 ± 0.2, 1.9 ± 0.22, and 3.4 ± 0.7 mg/L. Measured values were based on a time-weighted average of in- and out-water samples (Supplemental Information) (OECD 2012b). The solutions in each beaker were made fresh daily, and the water was renewed two hours after the first feeding daily.

### Analytical Chemistry

The analytical method used for analysis was adapted from USEPA Draft Method 1633 (USEPA 2021). To quantify PFOS, a 7 mL aliquot sample was diluted in the original collection vessel with 7 mL of methanol (MeOH). After the addition of MeOH, the samples were diluted as needed to fall within the instrument's linear range for PFOS. The internal standard used for quantitation was 13C8-PFOS (Wellington Laboratories, Ontario, Canada) and was present at 1 µg/L. The sample was in a solution of minimum 80:20 (MeOH:H_2_O) for analysis by triple quadrupole liquid chromatography tandem mass spectrometry (LC–MS/MS). All samples were analyzed using an Agilent 1290 Infinity Binary Pump LC coupled to an Agilent 6495B triple quadrupole MS/MS with Jet Streaming Technology and electrospray ionization (ESI) (Agilent, Santa Clara, California). Chromatographic separation was performed using an Agilent Poroshell 120 EC C18 column (2.1 × 100 mm, 1.9 µm). An Agilent Eclipse Plus C18 RRHD column (3.0 × 50 mm, 1.8 µm) was installed between the pump and autosampler to delay any potential contamination from mobile phases or inherently in the system. The background levels from the delay column were checked at the beginning of each sequence. Data acquisition was performed in dynamic multiple reaction monitoring (dMRM) mode using negative-mode ESI; 498.9 → 79.9 was the transition used for quantification and 498.9 → 98.8 was used for conformation. Chromatographic separation was achieved using a gradient elution with a flow rate of 0.35 mL/min. The analytical column was maintained at 40 °C throughout the run. The aqueous phase consisted of 2 mM ammonium acetate with 3% acetonitrile in MS grade water (A), and 100% MS grade acetonitrile (B). The injection volume was 5 *µ*L for this analysis.

### Food Preparation, Handling, and Ration Density Determinations

*Artemia* cysts were hatched in 1.75-L separatory funnel cones (Brine Shrimp Direct, Ogden, Utah) containing 30 ppt Crystal Sea® Marinemix, agitated by constant aeration supplied through a glass tube, and heated to approximately 23 °C by a 60-Watt light bulb. To collect *Artemia* for feeding, aeration was terminated and nauplii were allowed to settle to the bottom of the funnel for 10 min. Live *Artemia* were collected into a beaker by opening the separatory funnel valve, poured into a 150-micron nylon mesh net (excluding any unhatched cysts that settled to the bottom of the funnel), rinsed to avoid salt introduction into the test chambers, and resuspended in reverse osmosis water. The beaker of *Artemia* was agitated by a pipet in random directions to promote homogeneity and avoid higher densities in the middle of the beaker from circular stirring. *Artemia* density was determined by immediately subsampling 0.1 mL (collected by volumetric pipette) into a gridded counting petri dish with acetone. The amount of mL for each feeding was determined using Eq. (1):

1 $$\frac{0.1\, \text{mL}\, (\text{volume} \text { pipeted} \text{ in} \text{ counting} \text{ dish})}{\text{number} \text{ of } Artemia \text{ in} \text{ counting} \text{ dish}}=\frac{x\, (\text{mL} \text{ to} \text{ feed} \text{ per} \text{ beaker})}{Artemia \text{ ration} \text{ per} \text{ feeding}}$$

“*x*” was calculated by multiplying 0.1 mL by the *Artemia* ration per feeding and dividing by the number of *Artemia* in the counting dish. The resulting number was converted to microliters and pipetted into each beaker. The *Artemia* ration per feeding was calculated by dividing the total amount of *Artemia* to be fed per day, by the number of feedings (e.g., fish receiving 1000 *Artemia* daily received ≈ 500 *Artemia* in two separate feedings).

### Determining Individual Fish Weights

Prior to the start of both studies, 3 × 2 inch aluminum foil sheets were cut and placed in a 60 °C oven for three days and desiccator for one day to remove moisture and weighed to the nearest 0.001 mg using a microbalance (Sartorius Quintix125D-1S Analytical Semi-Micro Balance 60 g × 0.001 mg, Göttingen, Germany). At the end of the experiment, larval fish from each replicate were euthanized via tricaine mesylate (MS-222, Lot No. 12354) (Syndel, Ferndale, Washington). Excess moisture was removed by placing fish on a blotting pad, before being placed on a foil sheet (one sheet for each replicate) and put back in the oven for two days and desiccator for one day. The sheets containing dried larval fish were weighed, and the difference between that biomass and the initial empty sheet mass was determined to be the whole fish biomass in each replicate. All tests herein were acceptable per USEPA (2002a) test acceptability criteria, with 80% or greater survival in controls and an average dry weight per surviving organism in control chambers equal or exceeding 0.25 mg. Individual fish biomasses were calculated by dividing the biomass of the replicate by the number of fish placed in the sheet at the end of the experiment.

### Datal Analysis

Statistical analyses were performed and graphs were created in GraphPad Prism 8.0 (GraphPad Software LaJolla, California) for the control portion of the study and RStudio (v4.0.3., RStudio, Boston, Massachusetts) for the PFOS portion. In the control portion, a parametric one-way ANOVA using Bartlett’s test was employed to compare the individual biomass of each fish between treatments. From a Shapiro–Wilk test, it was found that the data were normally distributed, and transformations were not needed. Also, for the control feeding study, three separate three-way ANOVAs were performed between Groups 1, 2, and 3. Tukey’s multiple comparison test was used for post-hoc comparisons of means. The independent factors analyzed in the three-way ANOVAs were fish density, total *Artemia* provided, and feeding frequency, against the dependent variable of individual fish biomass. Statistical significance was determined at *α* = 0.05 for all analyses. The datasets generated and analyzed in the current study are available from the corresponding author upon reasonable request.

### Toxicological Endpoints

Time-weighted averages were calculated from measured PFOS concentrations and were used to determine toxicological endpoints and for statistical analyses. Since PFOS solutions were mixed daily and refreshed in the test vessels, time-weighted averages allow for a more representative estimate of the actual larval fish exposure (OECD 2012b). Toxicological endpoints were calculated using ToxCalc 5.0 (Tidepool Scientific Software, McKinleyville, California). The No Observable Effect Concentration (NOEC) and Lowest Observable Effect Concentration (LOEC) were determined by USEPA hypothesis testing methods (USEPA 2002a, 2002b; USEPA/USACE 1998). Data normality (Shapiro-Wilk Test), homogeneity (Bartlett’s Test), and treatment differences compared to the control (one-way ANOVA and Dunnett’s Method) were determined at the *α* = 0.05 level (one-tailed analysis). Survival data were arcsine-square-root transformed. Lethal median concentrations, LC_50_, and inhibition concentrations, IC, were calculated via the trimmed Spearman–Karber method and linear interpolation methods, respectively. Exposure response plots including 95% confidence intervals were generated using RStudio (v4.0.3., RStudio, Boston, Massachusetts).

## Results

### Control Study

#### Individual Biomasses One-Way ANOVA

**Fig. 1:**
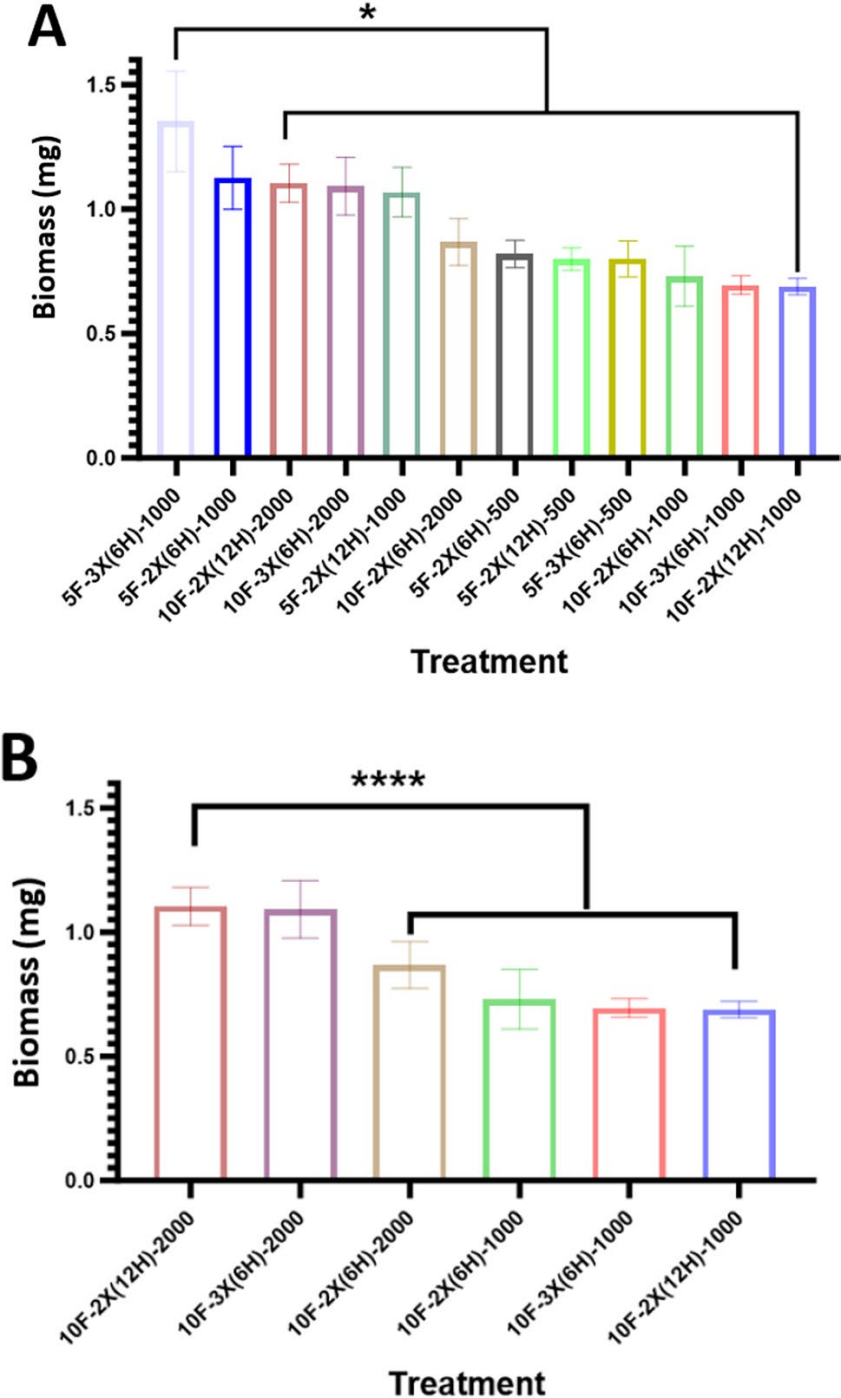
**A** Average individual fish biomasses for all treatments at both 5 and 10 fish/beaker density. **B** Average individual fish biomasses from each control feeding study, by treatment for the 10 fish/beaker density. Asterisks denote a significant difference in biomass between treatments (* = *p* ≤ 0.05; **** = *p* ≤ 0.0001)

Two fish densities (5 and 10 fish/beaker) were tested in the control study. Treatments 5F-3X(6H)-1000 and 5F-2X(6H)-1000 that contained 5 fish/beaker, resulted in the highest fish biomasses (Fig. 1A). Treatments 10F-2X(12H)-2000 and 10F-3X(6H)-2000, containing 10 fish/beaker, were the next two highest (Fig. 1A). The commonality between these four treatments is they were fed the maximum amount of *Artemia* daily (i.e., 2000 for beakers containing 10 fish and 1000 for beakers containing 5 fish). Figure 1B illustrates the significant biomass differences between beakers of 10 fish fed a high ration versus a low ration of *Artemia*. The three top HFR treatments of 2000 organisms have greater biomasses compared to the three LFR treatments fed 1000 *Artemia* daily (Fig. 1B).

### Effect of Ration, Time Interval and Number of Feedings Daily

In three separate three-way ANOVA analyses comparing Group 1, Group 2, and Group 3, the effects of fish density, total *Artemia* provided, and feeding frequency on fish biomass were examined. In the first analysis between Group 3 and Group 1, significant differences (*p* < 0.0001) of fish biomass in different treatments were found (Supplemental Fig. 1). Total *Artemia* fed accounted for 46% (Sum of Squares (SS) 3.861) of the total variation, feeding frequency for 8% (SS 0.6684), and fish density 6% (SS 0.5350) (total SS = 8.1926). The second analysis comparing Group 2 and Group 3, revealed significant differences between treatments (*p* < 0.0001) (Supplemental Fig. 2). Total *Artemia* fed accounted for 38% (SS 2.118) of the total variation, time of second feeding for 2% (SS 0.1147), and fish density 1% (SS 0.0746) (total SS = 5.42348). The third analysis comparing Group 2 and Group 1 also indicated significant differences (*p* < 0.0001) between treatments (Supplemental Fig. 3) and found total *Artemia* fed accounted for 52% (SS 5.092) of the total variation, feeding frequency for 2% (SS 0.2317), and fish density less than 1% (SS 0.0386) (total SS = 9.93898). The common theme across these analyses is that fish had greater biomass when fed a high ration of *Artemia.*

**Fig. 2:**
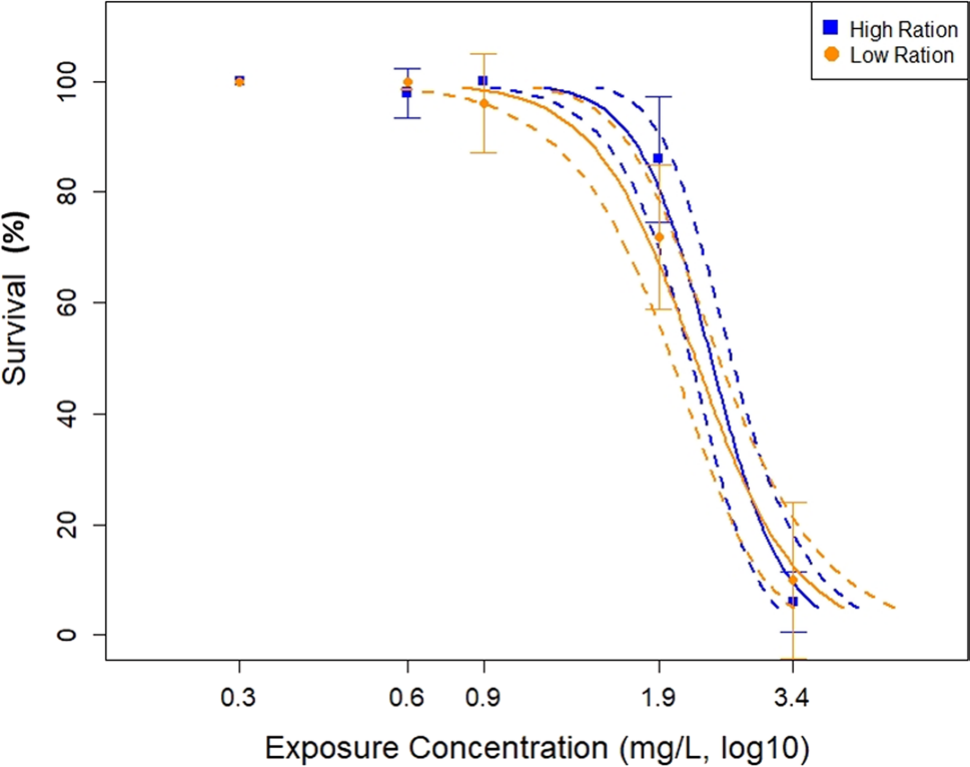
Exposure response plot for survival after a PFOS exposure in high feeding ration (blue) and low feeding ration (orange) treatments. Solid lines represent the generalized linear model fit of the data while dashed lines represent 95% confidence intervals. Error bars represent one standard deviation from the mean

**Fig. 3:**
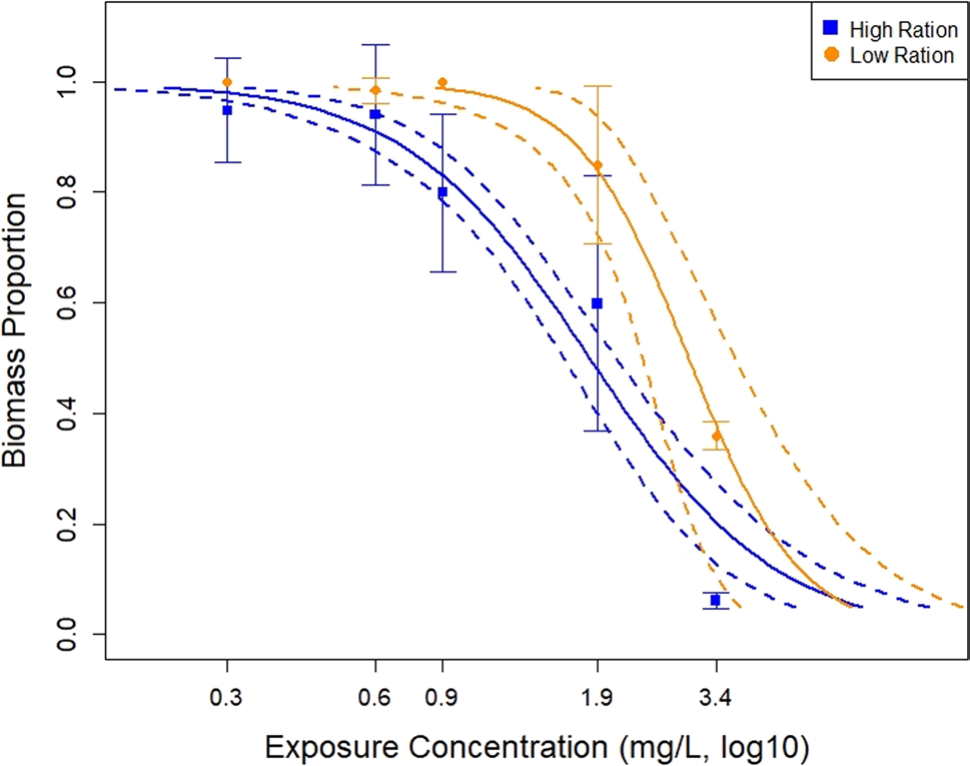
Exposure response plot for biomass in the high ration (blue) and low ration (orange) treatments for the PFOS exposure study. Solid lines represent the generalized linear model fit of the data while dashed lines represent 95% confidence intervals. Error bars represent one standard deviation from the mean

### PFOS Subchronic Exposure

#### Survival and Biomass Impacts in High Feeding Ration (HFR) and Low Feeding Ration (LFR) Treatments

The control subchronic portion of the study found that feeding ration and, secondarily, feeding frequency were the most impactful factors influencing fish survival and biomass. Our objective in this second phase was to examine the effects of those factors in the context of a subchronic exposure to PFOS. Survival NOEC, LOEC, and LC_50_ thresholds were calculated for the HFR and LFR treatments after a 7-day PFOS exposure (Table 2). The LFR treatment had marginally lower survival endpoints compared to the HFR treatment, indicating greater sensitivity of fish fed the LFR to the PFOS exposure (Table 2). However, when comparing the confidence intervals for the LC_50_ values, there is overlap between the HFR and LFR treatments, indicate lack of statistical significance (Table 2). Furthermore, graphics showing the survival curve’s overlap indicate that this endpoint is not statistically affected by feeding ration (Fig. 2). Overall, the different feeding rations during a PFOS exposure did not significantly impact the survival endpoint of *P. promelas*.

**Table 2:** Toxicological endpoints with 95% confidence intervals via ToxCalc software

		High feeding ration (HFR)		Low feeding ration (LFR)	
		Value (mg/L)	(Lower Limit, Upper Limit)	Value (mg/L)	(Lower Limit, Upper Limit)
Survival	NOEC	1.91	NA	0.89	NA
Survival	LOEC	3.42	NA	1.91	NA
Survival	LC_50_	2.44	(2.24–2.65)	2.25	(2.00–2.52)
Biomass	NOEC	1.91	NA	3.42	NA
Biomass	LOEC	3.42	NA	> 3.42	NA
Biomass	IC_25_	1.35	(0.51, 2.88)	2.64	NL
Biomass	IC_50_	2.80	NL	3.37	NL

*NA* not applicable, *NL* no limits

**Fig. 4:**
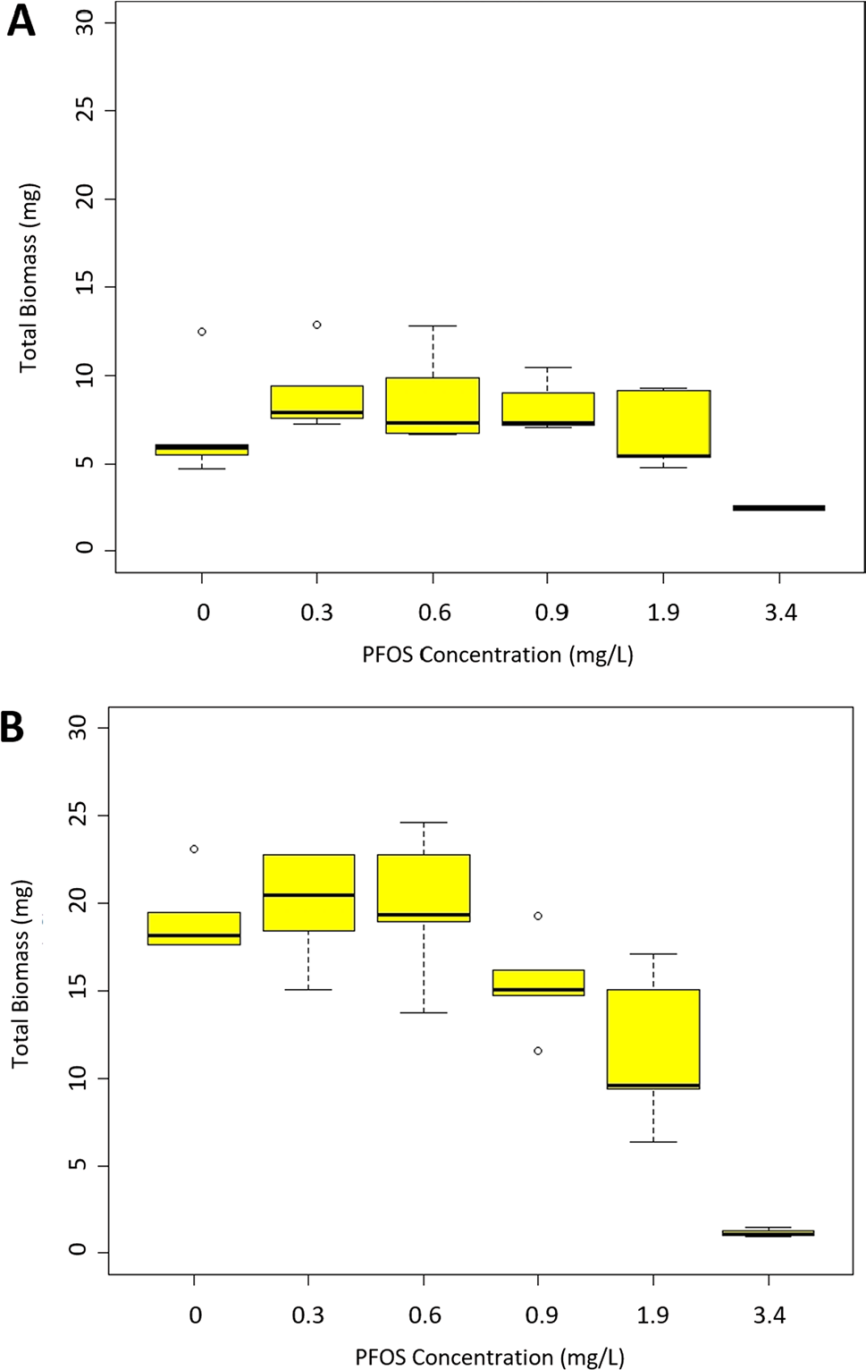
Box and whisker plots of fish biomass after the PFOS exposures. **A** summarizes biomass resulting from the low ration feeding treatment, while **B** represents biomass resulting from the high feeding ration. Boxes represent the 75th and 25th data percentiles, whiskers represent the 90th and 10th data percentiles and solid horizonal lines represent the median

The investigation into the impact of PFOS exposure on biomass reveals insights when considering the influence of dietary ration. Biomass NOEC, LOEC, IC_25_, and IC_50_ values were calculated for both treatment groups (Table 2). For purposes of statistical analyses, control biomass was set to 1, and constraining the higher values brings focus to the exposure response curve for the biomass endpoint (Fig. 3). In the low to middle concentrations, the HFR treatment demonstrates heightened sensitivity to the PFOS exposure. At higher concentrations, the HFR and LFR treatments biomasses converge (Fig. 3). Since the exposure–response curves were set to unity, we also present the magnitude of biomass variations in box and whisker plots (Fig. 4). Fish biomasses in the HFR treatment (Fig. 4B) are twice the mass as fish in the LFR treatment (Fig. 4A).

Further analyzation of this data was performed in GraphPad Prism. Two different 2-way ANOVAS analyzed the factors of PFOS concentration and feeding ration on the endpoints of survival and biomass. In the survival ANOVA, there was not a significant interaction between PFOS concentration and feeding ration (*p *= 0.0509), but PFOS was a significant factor (*p* < 0.0001) to survival. In the ANOVA examining the impact of PFOS concentration and feeding ration on the biomass endpoint, there was a significant interaction between the two factors (*p* = 0.0009). The difference of the means between the HFR and LFR groups was 7 (95% CI = 5.499 to 8.628). PFOS concentration and feeding ration accounted for 47% (SS = 1215) and 29% (SS = 748.3) of the total variation, respectively (total SS = 2605.2). Šídák’s multiple comparison’s test between the biomass of the HFR and LFR treatments in the PFOS exposure found HFR fish had significantly more biomass (*p* < 0.05) than LFR fish in all PFOS concentrations except the highest, 3.4 mg/L (Fig. 4).

## Discussion

The present study generated data to support logistically feasible and prescriptive guidance for the 7-day survival and growth *Pimephales promelas,* fathead minnow, subchronic toxicity bioassay (USEPA 2002a). Our primary objectives were to (1) determine how the independent factors of fish density, total *Artemia* provided, and number of daily feedings impacted the biomass of larval *P. promelas* and (2) implement a methodology that emphasized the most important factors in a case study with PFOS. The feeding methodology used in the PFOS portion of the study was designed to incorporate laboratory logistics, relevant considerations, and to not compromise water quality during testing. This study offers practical insights for consistent bioassay execution as per established guidance documents. The relevance and impact of this research are heightened by the acquisition of toxicity data for the emerging contaminant, PFOS.

We accomplished our first objective by determining that feeding ration and feeding frequency were the most important factors in the *P. promelas* 7-day subchronic bioassay. Specifically, a high ration of 2000 *Artemia* per 10 fish spread through 2 feedings (i.e., 1000 *Artemia* each) at an 8-h interval is sufficient for adequate survival and growth. Previous studies have evaluated effects of ad libitum feeding on growth of various fish species. Although the fish species are different, results from these studies corroborate the present study’s findings that two feedings daily is superior. Okomoda et al. (2019) examined feeding frequency in African catfish, *Clarias gariepinus*, and provided evidence of a threshold where more frequent feedings are critical for growth optimization. However, while Okomoda et al. (2019) found that five feedings per day of *Artemia* maximized growth in larval catfish, Dada and Olurewanju (2002) determined that growth was not significantly improved beyond three feedings per day. Similarly, fingerling carp, *Cyprinus carpio*, had larger biomasses when fed more than once daily (Biswas et al. 2006). In standard practice and based on documented survival, the longest gap of available live *Artemia* is between approximately 10pm to 8am (i.e., overnight), if the last feeding of the day is before the close of business. In our control study, a longer time gap without live *Artemia* was a detriment to final larval biomass in the groups fed two times per day with shorter intervals between feedings, so we knew in the PFOS portion, we would have to spread out the feedings.

Our second objective was accomplished by employing a high feeding ration and feeding frequency of twice daily in a PFOS exposure. This confirms that a HFR increases growth magnitude, even with a compound such as PFOS that was previously reported to inhibit growth in fathead minnows and zebrafish, *Danio rerio* (Shi et al. 2008; Du et al 2009; Suski et al 2020; Krupa et al. 2022). Environment Canada (2011) published guidelines for larval fathead minnows feeding in chronic tests and suggested that groups of ten fish be fed at least 1500, but preferably up to 2250 *Artemia* per day. Their recommendation is valid and corroborated our results. The mean biomass of fish fed the HFR was significantly greater (*p* < 0.05) that of fish fed the LFR in the PFOS study except at the highest concentration (Fig. 4). A LFR of 1000 *Artemia*/day may restrict growth potential of test fish. While the graphs show the HFR treatment had greater biomass compared to the LFR treatment, the NOEC and LOEC values indicate that the HFR biomass endpoint was more sensitive during the PFOS exposure. One explanation for this could be the HFR provided enough food for fish in the control and lower PFOS concentrations to grow at a greater rate than those in the higher concentrations, resulting in the large differences in biomass. A multiple comparisons test in the 2-way ANOVA for biomass did show that 3.4 mg/L was significantly (*p* < 0.05) lower in biomass than all concentrations, and that 1.9 mg/L was significantly lower than some as well. Whereas in the LFR treatment, there is not enough growth to provide a distinction of PFOS impacts.

Regarding survival, toxicological endpoints suggest the LFR had greater PFOS sensitivity, but overlapping confidence intervals indicate no significant difference between the LFR and HFR treatment. The 2-way ANOVA results also agree with the feeding ration having a low impact on survival; the PFOS concentration is the greatest determinant of survival. This finding indicates that fish in the HFR treatments were more tolerant to PFOS than those in the LFR treatments. The role that *Artemia* could play in reducing PFOS bioavailability, by mitigating toxic impacts, was not examined here, and may have aided in larval fish survival. Further testing through bioavailability studies would be warranted to determine that.

While it is important to observe a discernable effect in a toxicity test, it is also crucial to be realistic and consider the implications within the context of environmental concentrations. Environmental PFOS concentrations in surface waters are still being understood because data are limited and concentrations can vary widely based on location (e.g., being near a factory or point source). Observed concentrations currently range over 8 orders of magnitude but are generally between picograms and nanograms per liter (median concentration 3.6 ng/L) (Jarvis et al. 2021). However, our test concentrations of PFOS, ranging from 0.3 to 3.4 mg/L are realistic concentrations that have been observed in surface waters of some highly contaminated sites. Anderson et al. (2018) reported PFOS concentrations up to 8.7 mg/L in surface waters at Air Force bases contaminated with aqueous film-forming foam. When considering the potential ecological impacts of PFOS on growth and survival of larval fish, a comprehensive understanding of environmental factors and the risks posed by PFOS contamination is necessary.

Overall, the study results imply the 7-day *P. promelas* bioassay performs well in control experiments and chemical exposures when experimental replicates containing 10 fish that receive a total of 2000 *Artemia* nauplii daily. A twice daily feeding schedule with a minimum interval of 6-h achieves greater larval fish biomass. Provided that *Artemia* perish in freshwater, and larval fish will be less likely to eat dead *Artemia*, a larger interval between feedings would allow fish to always have the maximum amount of *Artemia* available. A thrice daily feeding schedule may be technically better to optimize fish growth; however, a laboratory worker would need work after normal business hours. Logistically, that feeding regime may be inconvenient, and the magnitude of improvement on test endpoints does not warrant the additional effort and cost. Ultimately, we encourage scientists to apply and report prescriptive daily feeding rations for future 7-day chronic *P. promelas* tests and to consider the impact of feeding methodologies on toxicological endpoints.

### Supplementary Information

Below is the link to the electronic supplementary material.

Supplementary file1 (PDF 73 KB)

Supplementary file2 (XLSX 29 KB)

## References

[CR1] Aderolu AZ, Seriki BM, Apatira AL, Ajaegbo CU (2010). Effects of feeding frequency on growth, feed efficiency and economic viability of rearing African catfish (*Clarias gariepinus*, Burchell 1822) fingerlings and juveniles. African J. Food Sci..

[CR2] American Society for Testing and Materials (2022). American Society for Testing and Materials Standard guide for conducting early life-stage toxicity tests with fishes (E1241–22). Annual Book of ASTM Standards.

[CR3] Anderson RH, Long G C, Porter, RC, Anderson J K (2018) Occurrence of select perfluoroalkyl substances at US Air Force aqueous film-forming foam release sites other than fire training areas: field validation of critical fate and transport properties. Perfluoroalkyl Substances in the Environment (pp. 353–372). 10.1016/j.chemosphere.2016.01.014 26786021

[CR4] Ankley GT, Kuehl DW, Kahl MD, Jensen KM, Linnum A, Leino RL, Villeneuve DA (2005). Reproductive and developmental toxicity and bioconcentration of perfluorooctanesulfonate in a partial life-cycle test with the fathead minnow (*Pimephales promelas*). Environ Toxicol Chem.

[CR5] Barbo N, Stoiber T, Naidenko OV, Andrews DQ (2023). Locally caught freshwater fish across the United States are likely a significant source of exposure to PFOS and other perfluorinated compounds. Environ Res.

[CR6] Bartlett AJ, De Silva AO, Schissler DM, Hedges AM, Brown LR, Shires K, Parrott JL (2021). Lethal and sublethal toxicity of perfluorooctanoic acid (PFOA) in chronic tests with *Hyalella azteca* (amphipod) and early-life stage tests with *Pimephales promelas* (fathead minnow). Ecotoxicol Environ Saf.

[CR7] Biswas G, Jena JK, Singh SK, Patmajhi P, Muduli HK (2006). Effect of feeding frequency on growth, survival and feed utilization in mrigal, *Cirrhinus mrigala*, and rohu, *Labeo rohita*, during nursery rearing. Aquaculture.

[CR8] Environment Canada (2011) Biological test method: Test of larval growth and survival using fathead minnows 2^nd^ Edition (EPS-1/RM/22). Method Development and Applications Unit Science and Technology Branch, Ottawa-Ontario: Environment Canada. https://publications.gc.ca/collections/collection_2011/ec/En49-7-1-22-eng.pdf. Accessed 2 April 2024

[CR9] Dada AA, Olarewaju O (2002) The Influence of feeding frequency on the growth and feed utilization of Catfish, *Clarias gariepinus* fry in outdoor concrete tanks. Biosci Res J, 14(4).

[CR10] Du Y, Shi X, Liu C, Yu K, Zhou B (2009). Chronic effects of water-borne PFOS exposure on growth, survival and hepatotoxicity in zebrafish: a partial life-cycle test. Chemosphere.

[CR11] Gust KA, Mylroie JE, Kimble AN, Wilbanks MS, Steward CS, Chapman KA, Moore DW (2023). Survival, growth, and reproduction responses in a three-generation exposure of the zebrafish (*Danio rerio*) to perfluorooctane sulfonate. Environ Toxicol Chem.

[CR12] Hayman NT, Rosen G, Colvin MA, Conder J, Arblaster JA (2021). Aquatic toxicity evaluations of PFOS and PFOA for five standard marine endpoints. Chemosphere.

[CR13] Jarvis AL, Justice JR, Elias MC, Schnitker B, Gallagher K (2021). Perfluorooctane sulfonate in US ambient surface waters: a review of occurrence in aquatic environments and comparison to global concentrations. Environ Toxicol Chem.

[CR14] Kasiri M, Farahi A, Sudagar M (2011) Effects of feeding frequency on growth performance and survival rate of angel fish, *Pterophyllum scalare* (Perciformes: Cichlidae). Veterinary Research Forum (Vol. 2, No. 2, pp. 97–102).

[CR15] Kikuchi K, Iwata N, Kawabata T, Yanagawa T (2006). Effect of feeding frequency, water temperature, and stocking density on the growth of tiger puffer, *Takifugu rubripes*. J World Aquaculture Soc.

[CR16] Krupa PM, Lotufo GR, Mylroie EJ, May LK, Gust KA, Kimble AN, Moore DW (2022). Chronic aquatic toxicity of perfluorooctane sulfonic acid (PFOS) to *Ceriodaphnia dubia*, *Chironomus dilutus*, *Danio rerio*, and *Hyalella azteca*. Ecotoxicol Environ Saf.

[CR17] Lall SP, Tibbetts SM (2009). Nutrition, feeding, and behavior of fish. Vet Clin North America: Exotic Animal Pract.

[CR18] Macorps N, Le Menach K, Pardon P, Guérin-Rechdaoui S, Rocher V, Budzinski H, Labadie P (2022). Bioaccumulation of per-and polyfluoroalkyl substance in fish from an urban river: occurrence, patterns and investigation of potential ecological drivers. Environ Pollut.

[CR19] Mohamed AM, Mohamed SA, Mostafa MM, Hamza ESM (2019). Impact of household cooking on release of fluorinated compounds PFOA and PFOS from Tefal coated cookware to foods. World J Adv Res Rev.

[CR20] Oakes KD, Sibley PK, Solomon KR, Mabury SA, Van Der Kraak GJ (2004). Impact of perfluorooctanoic acid on fathead minnow (*Pimephales promelas*) fatty acyl-coa oxidase activity, circulating steroids, and reproduction in outdoor microcosms. Environ Toxicol Chem: Int J.

[CR21] Okomoda VT, Aminem W, Hassan A, Martins CO (2019). Effects of feeding frequency on fry and fingerlings of African catfish *Clarias gariepinus*. Aquaculture.

[CR22] Organization for Economic Co-operation and Development (2012a) Organization for Economic Co-operation and Development Test No. 229: Fish Short Term Reproduction Assay OECD Guidelines for the Testing of Chemicals, Section 2. OECD Publishing, Paris. 10.1787/9789264185265-en

[CR23] Organization for Economic Co-operation and Development (2012b) Organization for Economic Co-operation and Development Test No. 211: *Daphnia magna* Reproduction Test OECD Guidelines for the Testing of Chemicals, Section 2. OECD Publishing, Paris. 10.1787/9789264185203-en

[CR24] Organization for Economic Co-operation and Development (2013) Organization for Economic Co-operation and Development Test No. 210: Fish, Early-life Stage Toxicity Test OECD Guidelines for the Testing of Chemicals, Section 2. OECD Publishing, Paris. 10.1787/9789264203785-en

[CR25] Seyyedsalehi MS, Boffetta P (2023). Per-and poly-fluoroalkyl substances (PFAS) exposure and risk of kidney, liver, and testicular cancers: a systematic review and meta-analysis. Med Lav.

[CR26] Shi X, Du Y, Lam PK, Wu RS, Zhou B (2008). Developmental toxicity and alteration of gene expression in zebrafish embryos exposed to PFOS. Toxicol Appl Pharmacol.

[CR27] Sontake AR, Wagh SM (2014). The phase-out of perfluorooctane sulfonate (PFOS) and the global future of aqueous film forming foam (AFFF), innovations in firefighting foam. Fire Eng.

[CR28] Suski JG, Salice CJ, Chanov MK, Ayers J, Rewerts J, Field J (2020). Sensitivity and accumulation of perfluorooctanesulfonate and perfluorohexanesulfonic acid in fathead minnows (*Pimephales promelas*) exposed over critical life stages of reproduction and development. Environ Toxicol Chem.

[CR29] Trier X, Granby K, Christensen JH (2011). Polyfluorinated surfactants (PFS) in paper and board coatings for food packaging. Environ Sci Pollut Res.

[CR30] United Sates Environmental Protection Agency/United States Army Corps of Engineers (1998) Evaluation of dredged material proposed for discharge in waters of the US-testing manual: Inland Testing Manual (EPA-823-B-987–004). Office of Water (4303T). https://apps.dtic.mil/sti/citations/tr/ADA338883. Accessed 10 April 10 2024

[CR31] United States Environmental Protection Agency (2002a) Short-term Methods for Estimating the Chronic Toxicity of Effluents and Receiving Waters to Freshwater Organisms (EPA-821-R-02–013). Office of Water (4303T). https://www.epa.gov/sites/default/files/2015-08/documents/short-term-chronic-freshwater-wet-manual_2002.pdf. Accessed 2 April 2024

[CR34] United States Environmental Protection Agency (2002b) Methods for Measuring the Acute Toxicity of Effluents and Receiving Waters to Freshwater and Marine Organisms (EPA-821-R-02–012). Office of Water (4303T). https://www.epa.gov/sites/default/files/2015-08/documents/acute-freshwater-and-marine-wet-manual_2002.pdf. Accessed 2 April 2024

[CR32] United States Environmental Protection Agency (2006) Fathead Minnow (*Pimephales promelas*) Larval Survival and Growth Toxicity Tests Supplement to Training Video (EPA-833-C-06–001). Office of Wastewater Management, Water Permits Division. https://www.epa.gov/sites/default/files/2016-02/documents/fathead_minnow_pimephales_promelas_larvel_survival_and_growth_toxicity_test_-_supplemental_video_training_guide.pdf. Accessed 2 April 2024

[CR33] United States Environmental Protection Agency (2021) Draft Method 1633 Analysis of Per- and Polyfluoroalkyl Substances (PFAS) in Aqueous, Solid, Biosolids, and Tissue Samples by LC-MS/MS (EPA 821-D-21–001). https://www.epa.gov/system/files/documents/2021-09/method_1633_draft_aug-2021.pdf. Accessed 2 April 2024

