# Supplementary material for: Feeding Ration Impacts Larval Pimephales Promelas 7-Day Subchronic Growth Endpoint: Case Study with Perfluorooctanesulfonic Acid
Source: Arch Environ Contam Toxicol. 2024 May 25;86(4):383–92. doi: 10.1007/s00244-024-01068-8 (PMC11142975; doi:10.1007/s00244-024-01068-8)
Supplement: Supplementary file 1 — Supplementary file1 (PDF 73 KB) [file 244_2024_1068_MOESM1_ESM.pdf]

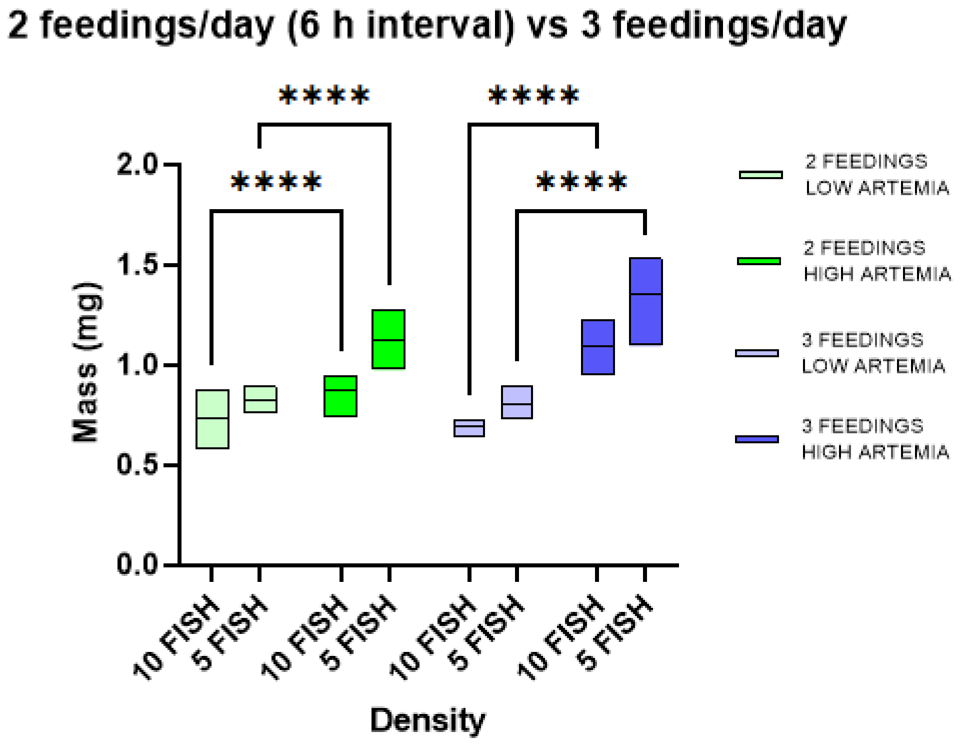

Supplemental Fig 1 Individual fish masses for Group 3 and Group 1. Brackets and asterisks denote significance between high and low ration (**** = $p < 0.0001$)

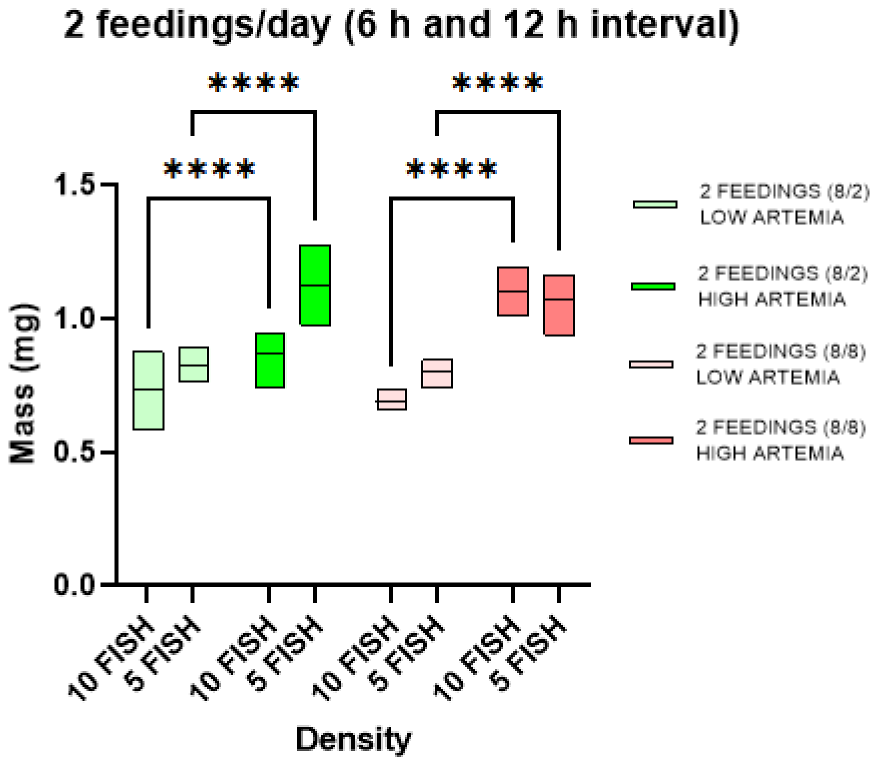

Supplemental Fig 2 Individual fish masses for Group 2 and Group 3. Brackets and the asterisks above denote significance in biomass between treatments (**** = $p < 0.0001$).

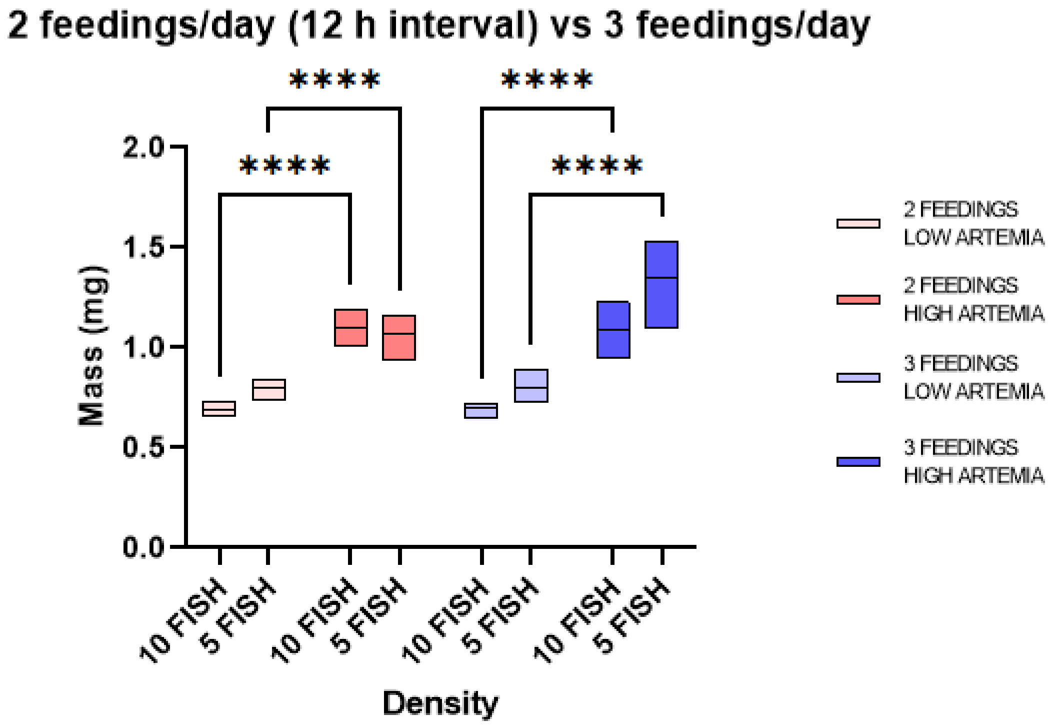

Supplemental Fig 3 Individual fish masses for Group 2 and Group 1. Brackets and the asterisks above denote significance in biomass between treatments (**** = $p < 0.0001$)
